# Prefrontal cortical deficits are a putative susceptibility factor for PTSD

**DOI:** 10.3389/fnbeh.2026.1750991

**Published:** 2026-03-04

**Authors:** Rebecca Nalloor, Khadijah Shanazz, Almira Vazdarjanova

**Affiliations:** 1 VA Research Service, Charlie Norwood VA Medical Center, Augusta, GA, United States; 2 Department of Pharmacology and Toxicology, Medical College of Georgia at Augusta University, Augusta, GA, United States; 3 Department of Neuroscience and Regenerative Medicine, Medical College of Georgia at Augusta University, Augusta, GA, United States

**Keywords:** *Arc*, catFISH, cognitive function, *Homer 1a*, IEG, resilience, RISP model

## Abstract

**Introduction:**

Only a subset of people who experience a traumatic event develop Post-Traumatic Stress Disorder (PTSD) suggesting that there are susceptibility factors influencing PTSD pathophysiology. While post trauma sequelae factors are extensively studied, susceptibility factors are difficult to study and therefore poorly understood. To address this gap, we previously developed an animal model - Revealing Individual Susceptibility to PTSD-like phenotype (RISP). RISP allows studying susceptibility factors by identifying, before trauma, male rats that are likely to develop a PTSD-like phenotype after trauma. Hypofunctioning prefrontal cortex (PFC) has been reported in people with PTSD, however, it is unclear if it is a susceptibility factor, sequalae factor, or both. Here we tested the hypothesis that male rats classified as Susceptible with RISP will have altered medial prefrontal cortical (mPFC) function prior to a PTSD-inducing trauma.

**Methods:**

Experiment 1: Susceptible and Resilient male rats classified with RISP performed spatial exploration and were sacrificed immediately to assess neuronal expression of plasticity-related immediate early genes (Arc and Homer1a) in the medial PFC (mPFC). Experiment 2: Cognitive performance of Susceptible and Resilient rats was evaluated on an attentional set shifting task. Experiment 3: We also analyzed pre-trauma cognitive performance scores of a small group of male military personnel some of whom developed PTSD post-trauma.

**Results:**

Experiment 1: Susceptible rats showed altered expression of plasticity-related immediate early genes in the Prelimbic and Infralimbic subregions of the mPFC following spatial exploration. Experiment 2: Susceptible rats showed deficits in attentional set shifting task only when task demands increased. Experiment 3: Male military personnel who developed PTSD post-trauma showed pre-trauma cognitive deficits in a task involving the PFC.

**Discussion:**

Susceptible rats showed mPFC deficits both at the cellular and behavioral level before PTSD-inducing trauma. Combined with the findings from the human data, these results support the hypothesis that mPFC deficits in males exist before trauma and thus are a putative susceptibility factor for PTSD. Whether these deficits are a bona fide susceptibility factor will be determined in future studies by testing if enhancing mPFC function in susceptible individuals before trauma will confer resilience to developing PTSD. Building resilience is crucial for minimizing the number of people suffering from PTSD, given that it is difficult to treat and treatments are resource intensive and benefit only a subpopulation of people suffering from PTSD.

## 1 Introduction

Post-Traumatic Stress Disorder (PTSD) is a complex mental disorder that develops in a subset of people exposed to a traumatic event and results in long-lasting emotional and functional impairments (American Psychiatric Association [APA], 2000). Individual susceptibility to developing PTSD has long been acknowledged (Yehuda et al., 2000; Gilbertson et al., 2002; Childress et al., 2013; Logue et al., 2018), yet it is poorly understood. To facilitate investigations into individual susceptibility, we have previously postulated a unifying framework of risk, susceptibility and sequelae factors (Alexander et al., 2020)

We define susceptibility factors as those aspects that are present and can be identified before trauma and manipulated such that they affect the onset and progression of PTSD after trauma (Alexander et al., 2020). To identify and study susceptibility factors, we developed an animal model, RISP, (Revealing Individual Susceptibility to PTSD-like phenotype). Using RISP, one can classify male rats before trauma as Resilient or Susceptible to developing a PTSD-like phenotype based on *a priori* set of criteria of acoustic startle response and anxiety-like behaviors 4 days after exposure to a mild stressor which does not induce fear conditioning. These are temporary changes in behavior in response to the mild stressor that return to baseline if the animals do not subsequently experience a traumatic event. If however, they do experience a traumatic even (strong foot shock), Susceptible, but not Resilient, rats display a PTSD-like phenotype with impaired fear extinction and sustained increase in acoustic startle response (Nalloor et al., 2011).

Using the RISP model in conjunction with a sensitive cellular imaging technique called *Arc/Homer1a* catFISH (cellular compartment analysis of temporal activity by fluorescent *in situ* hybridization using *Arc* and *Homer 1a*) we previously reported a putative susceptibility factor- impaired hippocampal learning-induced plasticity before trauma (Nalloor et al., 2014). During spontaneous spatial learning Susceptible, compared to Resilient, rats have impaired expression of immediate-early genes (IEGs) in the ventral/temporal hippocampus. Susceptible rats also showed altered pattern of IEG expression in the dorsal/septal hippocampus during the same low stress event. These differences were detected before the animals experienced a PTSD-inducing traumatic event suggesting that altered hippocampal function is a putative susceptibility factor. Such findings are consistent with reports in humans where hippocampal structural and functional deficits before trauma have been associated with increased severity of PTSD (Gilbertson et al., 2002; Gilbertson et al., 2006; Hayes et al., 2011; Xie et al., 2018).

Other brain regions functionally related to the hippocampus such as the medial prefrontal cortex (mPFC) and amygdala have also been shown to have structural and functional alterations in people with PTSD (Milad et al., 2009; Pitman et al., 2012; Harnett et al., 2020; Xiao et al., 2022). It is unclear, however, if these alterations exist before trauma and thus contribute to susceptibility to PTSD or develop because of the trauma. We hypothesize that these alterations exist before trauma and thus, may be putative susceptibility factors based on the evidence that susceptible male rats have increased inflammation in the mPFC and that inflammation disrupts neuronal function (Shanazz et al., 2023; Tastan and Heneka, 2024). If true, this will suggest that susceptibility emerges in a network of brain regions functionally related to the hippocampus rather than just the hippocampus.

We tested the hypothesis that mPFC has altered function in Susceptible compared to Resilient rats before PTSD-inducing trauma by examining plasticity in the Prelimbic (PL) and Infralimbic (IL) regions of the mPFC induced by a non-traumatic event using *Arc/Homer 1a* catFISH (Vazdarjanova et al., 2002; Vazdarjanova et al., 2006). For rigor, we used tissue from the same animals in which we had demonstrated alterations in *Arc/Homer 1a* expression in the hippocampus, but not the basolateral amygdala, during spatial exploration. This task was chosen to assess brain function under relatively non-stressful conditions to eliminate the possibility that the examined IEG expression is due to processing of a potentially traumatic event that can lead to PTSD-like behavior. Evidence in support of this hypothesis will satisfy the first step in identifying mPFC deficit as a susceptibility factor, i.e., it is present and identifiable before trauma.

**FIGURE 1:**
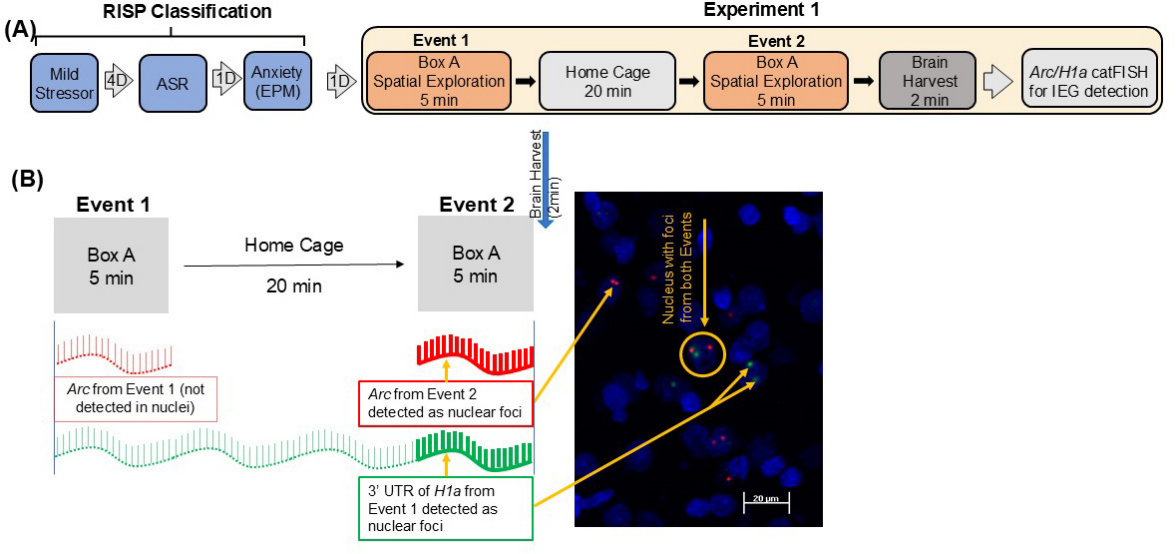
**(A)** RISP classification and design of Experiment 1. **(B)** Schematic representation of *Arc/Homer1a* catFISH with a representative image from IL–Nuclei (blue), *Arc* (red), *H1a* (green).

*Arc* (activity-regulated cytoskeleton-associated protein) and *Homer1a* (*H1a*) are effector IEGs that are very low at baseline and are coordinately expressed during learning (Steward and Worley, 2001; Bottai et al., 2002; Vazdarjanova et al., 2002; Bramham et al., 2008; Bramham et al., 2010). They are reliable markers of plasticity, not solely neuronal activity (Guzowski et al., 2006; Carpenter-Hyland et al., 2010) and are necessary for memory consolidation (Fagni et al., 2002; Ramírez-Amaya et al., 2005; Szumlinski et al., 2006; Bramham et al., 2010). Furthermore, *Arc* expression reliably predicts the pattern of plasticity assessed electrophysiologically a day after learning (Guzowski et al., 2006; Carpenter-Hyland et al., 2010). Additionally, *Arc* and *H1a* mRNA have different lengths such that, using a full-length Arc probe and a similar sized probe targeting the 3’ UTR of *H1a*, it is possible to dissociate neuronal ensembles that have initiated plasticity during two events experienced 20–30 min apart. Neurons with *H1a* foci of transcription mark those responding to the first event and the ones with *Arc* foci mark neurons responding to the second event. Neurons with both *H1a* and *Arc* foci have responded to both events (Vazdarjanova et al., 2002; Chawla et al., 2004; Nalloor et al., 2012; Nalloor et al., 2014; Figure 1B). One advantage of *Arc/H1a* catFISH is that the dissociation of the neuronal ensembles responding to these events can be done across multiple brain regions in the same animals. Combined with the RISP model, *Arc/H1a* catFISH allowed us to examine whether relative plasticity across different brain regions is altered in Susceptible compared to Resilient rats.

We also hypothesized that any differences in plasticity between Resilient and Susceptible rats will also be detected functionally with appropriate cognitive tests. Attentional Set-Shifting is a task that is commonly used to test cognitive flexibility in animals (Heisler et al., 2015; Britten et al., 2023). Impairments in this task are associated with structural changes and functional deficits in the mPFC (Birrell and Brown, 2000; Bissonette et al., 2013; Chen et al., 2023). Therefore, we tested whether the Resilient and Susceptible rats differ on their performance on the Attentional Set-Shifting task.

To begin evaluating the translational relevance of these hypotheses, we compared pre-deployment performance on a battery of cognitive tests of military personnel who developed post-deployment PTSD and those who did not. We hypothesized that susceptibility factors also exist in humans and can be functionally detected before a PTSD-inducing trauma.

## 2 Materials and methods

### 2.1 Subjects

#### 2.1.1 Animal

Two-three-month-old, adult (250–300 g) male Sprague-Dawley rats (Charles River Laboratories Inc., Wilmington, MA) were double housed on a 12 h light/dark cycle (lights on at 7:00 a m) with food and water freely available except during Experiment 2 (Attentional set shifting) when they were food restricted (Figure 3A). Testing was performed between 9:00 a.m. and 5:00 p.m. All behavioral procedures were approved by the Institutional Animal Care and Use Committee (IACUC), Augusta University and Charlie Norwood VA Medical Center, Augusta, GA.

#### 2.1.2 Human

De-identified pre-deployment ANAM (Vista Life Sciences, Inc., Englewood, CO) data and post deployment PTSD status from a small group of male (age: 23–58 years) active-duty military personnel was obtained (Fort Gordon, Augusta, GA). The study involving human participants was reviewed and approved by the Institutional Review Board at Eisenhower Medical Center, Fort Gordon, Augusta GA. Written informed consent to participate in the study was provided by the participants.

### 2.2 Rodent behavioral procedures

The number of rats per group is listed under each experiment and in each figure. All rodents were handled for 2–3 min for 3 consecutive days, starting 3 days after arrival. Behavioral testing began after handling. All behavioral procedures were approved by the Institutional Animal Care and Use Committee (IACUC), Augusta University and Charlie Norwood VA Medical Center, Augusta, GA.

### 2.3 RISP animal model

The RISP (Revealing Individual Susceptibility to a PTSD-like Phenotype) animal model of PTSD is described in detail in Nalloor, 2011 and Alexander 2020 (Nalloor et al., 2011; Alexander et al., 2020). Briefly, 4 days after a brief exposure to a mild stressor (cat hair), rats were tested for their acoustic startle response (ASR) and anxiety-like behavior on the elevated plus maze (EPM) (Figure 1A). Using *a priori* set criteria, they were classified as:

1. Resilient (Res)—low startle (average ASR and more than 7 of 15 individual ASR smaller than the group average ASR) and low anxiety-like behavior (at least 1 entry into the open arm of the EPM).
2. Susceptible (Sus)—high startle (average ASR and 6 or more of 15 individual ASR greater than the group average ASR) and high anxiety-like behavior (no entries into the open arms of the EPM).
3. Intermediate- Animals meeting neither set of criteria; excluded from further analysis.

Important features of RISP classification:

#### 2.3.1 The mild stressor

Is a brief (3 min) exposure to a ball of cat hair infused with 100 mcl cat urine (CH). Although stressful, (elevates corticosteroid levels in the blood) it is not a traumatic stimulus. Exposed animals do not show conditioned fear (freezing) to the chamber the following day, as they do the day after receiving foot-shocks in a novel place (contextual fear conditioning, CFC). CH is necessary to reveal susceptibility—without it virtually no animals can be identified as Susceptible before trauma even when using the same classification criteria. Additionally, behavior during the CH exposure cannot predict the post-trauma behavior. The changes in behavior in response to CH returns to baseline without a subsequent traumatic event.

CH contributes to the face validity of the model as it is consistent with studies showing that probability for PTSD is higher in professions with high level of unpredictable stress.

#### 2.3.2 Identifying susceptibility

Since the mild stressor is necessary but not sufficient to reveal susceptibility, the RISP model includes testing ASR and avoidance of the Open arms in the EPM. Importantly, performance on neither of them alone can predict the complex post-trauma PTSD-like phenotype.

#### 2.3.3 Identified susceptibility reliably predicts post-trauma phenotype

Sus rats have impaired fear extinction and trauma-induced sustained (> 2 weeks post-trauma) increase in ASR. Res rats however have steep extinction curves and maintain low ASR.

## 3 Experiment 1

The design of Experiment 1 is summarized in Figure 1A. The behavior of these animals during classification was previously reported in Nalloor et al. (2014). Rats classified as Resilient (*n* = 8) or Susceptible (*n* = 6) were allowed to explore a novel box (50 cm × 10 cm × 19 cm) twice for 5 min, 20 min apart (Event 1 and Event 2, respectively). The rats were in their home cages during the 20 min between the Events. Fear behavior and locomotion were assessed. Freezing, a measure of fear behavior, was defined as lack of all movement except for respiration. Number of Crossings, a measure of locomotor activity, was defined as all four paws and the base of the tail crossing over the midline of the box. Immediately after Event 2, rats were quickly anesthetized (< 1.5 min) and decapitated. Caged control (*n* = 3) rats were removed from the cages either before the first or second behavioral event and were immediately anesthetized for brain harvesting and assessing of baseline *Arc/H1a* expression.

### 3.1 Brain harvesting

The brains were rapidly harvested and flash-frozen in 2-methyl butane (time from initiation of anesthesia to freezing of the brain < 3 min). They were stored at −80°C until further processing.

### 3.2 *Arc/Homer1a* catFISH

The right hemisphere of brains from Res and Sus rats were blocked together in freezing medium with a maximum of 6 brains per block. Twenty-micron thick coronal sections were cut on a cryostat and mounted on glass slides. The slides were stored at −20°C or −80°C until further processing. The slides were processed for *Arc/Homer1a* catFISH as described by Guzowski and Worley (2001), Nalloor et al. (2012), Nalloor et al. (2014), and Dixon-Melvin et al. (2022) to detect transcription foci of *Arc* and *Homer1a (H1a)*. Briefly, after fixing in 4% paraformaldehyde and permeabilizing the tissue in 1:1 acetone/methanol, a fluorescein-labeled full-length *Arc* and digoxigenin-labeled *H1a* antisense riboprobe, targeting the 3’ UTR of *H1a* mRNA, were applied and the slides incubated overnight at 56°C. Following hybridization, slides underwent stringency washes, endogenous peroxidase quenching and blocking for non-specific antibody binding. The digoxigenin tag was detected sequentially with horseradish peroxidase-conjugated anti-digoxigenin antibody (Fab fragments, Roche Diagnostics, Indianapolis, IN) and tyramide amplification reaction with SuperGlo Green (Fluorescent Solutions, Augusta, GA). Following a second peroxidase quenching step, the fluorescein tag was revealed with horseradish peroxidase-conjugated anti-fluorescein antibody (Roche) and tyramide amplification reaction with SuperGlo Red (Fluorescent Solutions).

Riboprobes were generated using commercial transcription kits (Ambion MaxiScript, Life Technologies, Grand Island, NY; or AmpliScribe; Epicentre Biotechnologies, Madison, WI) and digoxigenin-UTP or fluorescein-UTP RNA labeling mixes (Roche). Coverslips were mounted and nuclei were counterstained using Vectashield Mounting Medium with DAPI (Vector Laboratories, Burlingame, CA).

### 3.3 Image acquisition and stereological analysis

Mosaic (850 × 650 μm) image stacks from Prelimbic (PL) and infralimbic (IL) regions of the medial prefrontal cortex (mPFC) were collected from at least 2 different slides per animal using a 20x objective on a Zeiss AxioImager/Apotome system. The mPFC (3.72–2.76 mm anterior to Bregma) was identified based on Figures 9–12 of The Rat Brain by Paxinos and Watson (Paxinos and Watson, 2006).

Unbiased stereological cell counting and classification were performed as follows: (1) Neuron-like cells in the regions of interest were segmented by using an optical dissector method (West, 1999) with the gene channels turned off to prevent selection bias; (2) segmented neurons were classified using Zeiss AxioVision imaging software. Putative glial cells (those with small, intensely, and uniformly stained nuclei) were excluded from the analysis. With the gene channels turned on, segmented neurons were classified as *Arc* + , *H1a* + or *Arc/H1a* + depending on whether they had foci of transcription for *Arc*, *H1a*, or both, respectively. Foci were quantified only when they were visible on 3 or more consecutive planes. Cells without any foci were classified as Negative. For purposes of clarity, we refer to all *H1a* + cells as an “activated ensemble” that initiated plasticity-related IEG expression during Event 1, and all *Arc* + cells as an activated ensemble from Event 2.

**FIGURE 2:**
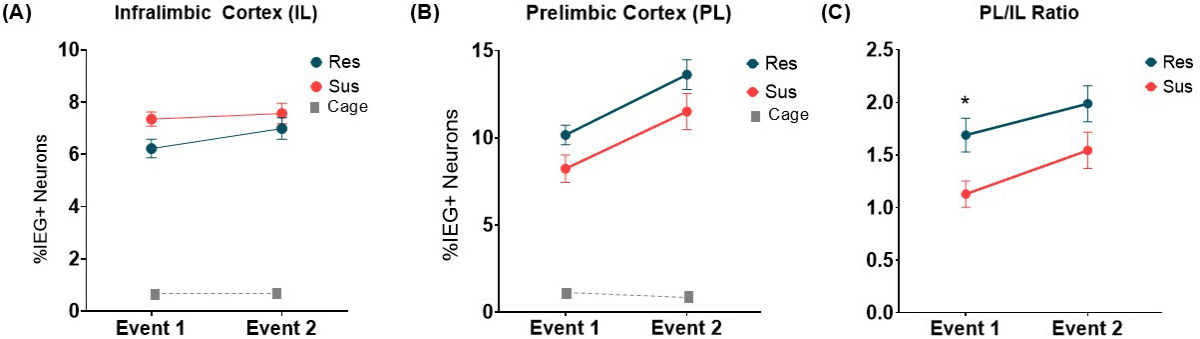
*Arc* and *H1a* expression in the mPFC of Resilient (Res, *n* = 8) and Susceptible (Sus, *n* = 6) rats during Event 1 and Event 2 in the **(A)** IL, **(B)** PL, and **(C)** The Pl/IL ratio of activated ensembles. *p = 0.024 (Caged, *n* = 3).

A schematic representation of the time course of Events in Experiment 1 and the corresponding expression of *Arc* and *H1a* are shown in Figure 1B. The results from Experiment 1 are shown in Figure 2.

## 4 Experiment 2

**FIGURE 3:**
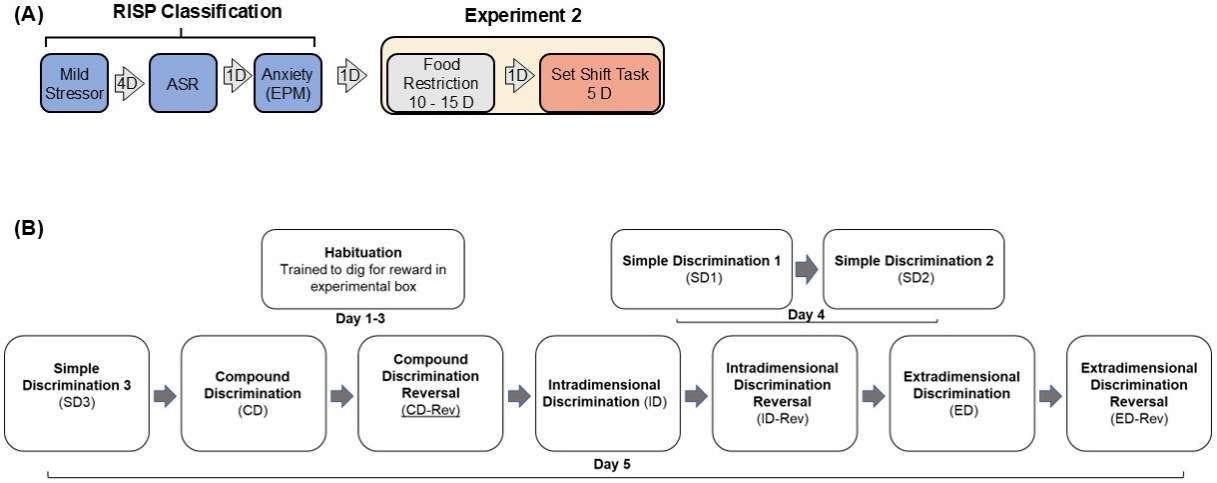
**(A)** RISP classification and design of Experiment 2. **(B)** Details of the Attentional Set-Shifting task.

The experimental design is summarized in Figure 3A. Similar to Experiment 1, rats were classified using the RISP model. Resilient (*n* = 11) and Susceptible (n = 9) rats were food restricted to maintain their body weight at ∼85% of pre-restriction weight.

Attentional Set-Shifting task: Details of the order of discrimination tasks are provided in Figure 3B.

### 4.1 Habituation

On Days 1–3, rats were habituated to a 30 × 25 × 30 cm chamber containing two cups (5 cm diam. × 4 cm deep) inserted into the floor. The cups contained either a digging medium alone (when habituating to a digging medium) or a digging medium and a scent (when habituating to scents). The rats were trained to dig in the cups with various scented and unscented media for a buried food reward (1/2 of a Froot Loops cereal piece). Each animal was exposed to all scents and media, one at a time. Previous screenings of the selected media and scents had shown no preference or discrimination against any of the selected media or scents.

### 4.2 Training and testing

**FIGURE 4:**
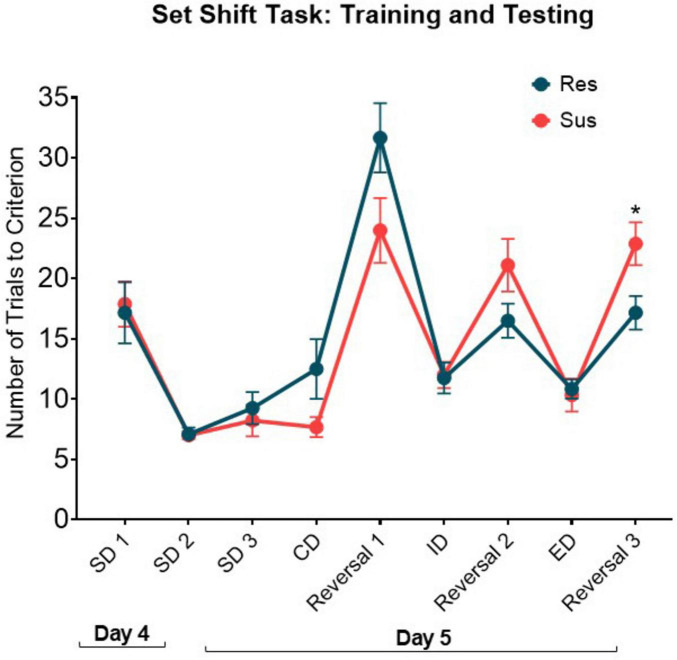
Performance of Resilient (Res, *n* = 11) and Susceptible (Sus, *n* = 9) rats on the training (day 4) and testing day (day 5) of Attentional Set-Shifting task. RM-ANOVA of the number of trials to criterion for each of the discriminations. SD, Simple Discrimination; CD, Compound Discrimination; ID, Intradimensional Discrimination; ED, Extradimensional Discrimination. *p = 0.022.

On days 4 and 5, each animal was placed into the chamber for up to 1 min per trial and allowed to dig in one of the two cups for the food reward (1/4 of a Froot Loops piece). Only one of the two cups (left or right) was baited with a food reward according to the training outline presented in Table 1. Trials were scored as “correct” if the animal chose to dig in the baited cup. After retrieving the food reward or after they stopped digging in the incorrect cup, animals were removed and placed into a holding cage between trials. Each discrimination task ended when the animal performed 6 consecutive correct trials. The length of each trial and number of trials to reach criterion were recorded. The presentation of the reward was pseudorandomized for right/left placement and medium/scent combination. The list of media and scents at each training stage is provided in Table 1 (the rewarded/baited items for each stage are in bold). For consistency, all rats were presented with the same set and order of discriminations, as described in Table 1. Results for Experiment 2 are shown in Figure 4.

**TABLE 1:** Set shifting media and scents.

	Task	Media		Scent	
Day 1	SD1	Corn cob bedding		**Basil**	Nutmeg
	SD2	**Corn cob bedding**	Paper pellet bedding	–	–
Day 2	SD3	Wood shavings		**Coffee**	Cinnamon
	CD	Paper pellet bedding	Sawdust	**Coffee**	Cinnamon
	CD-R	Paper pellet bedding	Sawdust	Coffee	**Cinnamon**
	ID	Corn cob bedding	Plastic beads	**Mint**	Cocoa
	ID-R	Corn cob bedding	Plastic beads	Mint	**Cocoa**
	ED	**Wood shavings**	Sand	Cumin	Oregano
	ED-R	Wood shavings	**Sand**	Cumin	Oregano

Bold represents rewarded items.

## 5 Human data

**FIGURE 5:**
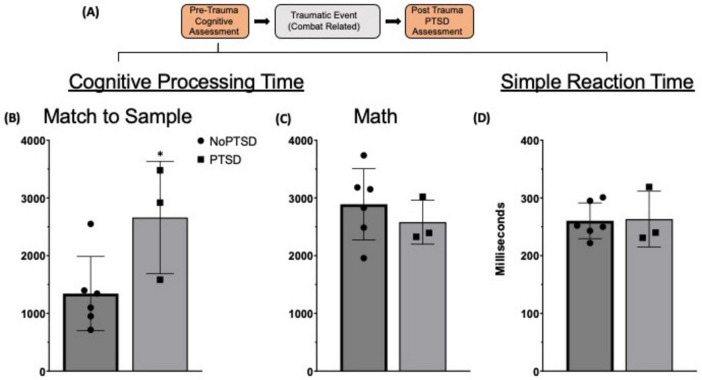
Pre-deployment cognitive performance of military personnel with post-deployment PTSD status **(A)** Timeline of key events reflecting the point at which the presented data was collected. (**B–D**) Pre-trauma ANAM data. *p = 0.042.

The timepoints of collection of the deidentified human data are provided in Figure 5A. The pre-trauma cognitive assessment was performed using ANAM (Automated Neuropsychological Assessment Metrics, Vista Life Sciences, CO), a battery of computerized cognitive tests that also evaluates simple reaction time at the first and second half of the battery. The ANAM data was collected before combat exposure. At this time, none of the subjects met the criteria for PTSD diagnosis. Subsequently, all subjects encountered severe combat related traumatic events that resulted in TBI. Following this trauma, some developed PTSD (PTSD; *n* = 3) and some did not (NoPTSD; *n* = 6). The PTSD evaluation and diagnosis were performed by qualified clinicians at Eisenhower Army Medical Center, Augusta, GA.

Both NoPTSD and PTSD groups were compared on the following outputs of their pre-deployment ANAM: Simple Reaction Times, Accuracy, Throughput and Reaction Time for each cognitive test. In order to normalize for possible differences in individual reaction time, we calculated Cognitive Processing Time:

Cognitive Processing Time = Reaction Time for test – (Simple Reaction Time 1 + Simple Reaction Time 2)/2

## 6 Statistical analyses

Two-way repeated measures ANOVA with Group and Event (Figure 2) or Task (Figure 4) as independent variables was used to compare IEG expressing ensembles or number of trails to criterion, respectively. Statistically significant main effects were followed up by Fisher’s LSD *post hoc* for between group comparisons and one-way ANOVA for within group comparisons. The difference between groups in the ratio of ensemble sizes (Event 2/Event1) was evaluated using one group *t*-test with a hypothesized difference of 1. In the human dataset, one-way ANOVAs were used to compare performance of No PTSD and PTSD groups in the different cognitive tests (Figures 5B–D) (GraphPad 10). Differences were considered statistically significant at *p* < 0.05.

## 7 Results

### 7.1 Resilient and Susceptible rats have different patterns of *Arc/Homer1a* expression in the mPFC in response to spatial exploration

Figure 1A illustrates the RISP model and design of Experiment 1. Illustration of IEG expression induced by Event 1 and Event 2 is shown in Figure 1B. The behavior of these animals during classification was previously reported in Nalloor et al. (2014). Here, one of the Resilient rats from the original data set was excluded due to damage to the mPFC during tissue processing. The *Arc/Homer1a* results reported here are from 8 Resilient and 6 Susceptible rats.

During the two spatial exploration events (Event 1 and Event 2), all animals explored the box well. There were no differences between Resilient and Susceptible rats in the Number of Crossings [Group effect: *F*(1, 12) = 0.052, *p* = 0.823, β = 0.055 and no Group × Event interaction: *F*(1, 12) = 2.260, *p* = 0.159, β = 0.271]. None of the rats displayed freezing > 5%. Despite similar behavior, differences were detected between groups in mPFC IEG ensembles responding to these events (Figures 2A–C). This data is based on an average cell count of 675 per animal/per brain region.

In IL (Figure 2A), there was an overall increase in ensemble size during Event 2 [Event effect: *F*(1, 12) = 6.212, *p* = 0.028, β = 0.628 with no Group effect: *F*(1, 12) = 2.356, *p* = 0.151, β = 0.281 and no Group × Event interaction: *F*(1, 12) = 1.955, *p* = 0.187, β = 0.240]. This ensemble size difference was driven by the Res group as the ratio of Event 2/Event 1 was significantly different from 1 only for the Res group (*p* = 0.026; Sus: *p* = 0.518). Baseline *Arc* and *H1a* expression in caged controls was < 1%. Additionally, the % double labeled cells, in the Sus rats was slightly higher than the Res rats [*F*(1, 12) = 4.293, p = 0.060, β = 0.469].

Similarly, in PL (Figure 2B), there was an increase in ensemble size during Event 2 [Event effect: *F*(1, 12) = 28.158, *p* = 0.0002, β = 0.999]. There was also a nearly significant Group effect [*F*(1, 12) = 4.478, *p* = 0.056, β = 0.485] with no Group × Event interaction [*F*(1, 12) = 0.019, *p* = 0.892, β = 0.052]. Furthermore, both groups showed increased ensemble size: Event 2/Event 1 was significantly > 1 for both Res and Sus groups (*p* < 0.0001). Baseline *Arc* and *H1a* expression in caged controls was < 1%. However, unlike IL, the% double labeled cells in the Sus rats were significantly lower [*F*(1, 12) = 6.834, *p* = 0.023 β = 0.672]. Overall, these findings point to a difference in pattern of IEG expression between the Sus and Res rats. We further evaluated this difference in the pattern of IEG expression between PL and IL in Res and Sus rats by comparing their PL/IL ratio. The PL/IL ratio increased for both groups during the second exploration [Event effect: *F*(1, 12) = 10.13, *p* = 0.008, β = 0.844]. This increase was different between groups such that the PL/IL ratio was significantly higher in the Res rats compared to the Sus rats [Group effect: *F*(1, 12) = 6.11, *p* = 0.029, β = 0.621] with no Group x Event interaction [*F*(1, 12) = 0.27, *p* = 0.610, β = 0.076]. Res rats had a bigger PL ensemble size than IL during both Event 1 (PL/IL = 1.69, *p* = 0.004) and Event 2 (PL/IL = 1.99, *p* = 0.0007), while in Susceptible rats this was true only for Event 2 (PL/IL = 1.54, *p* = 0.025). In Sus rats PL and IL ensemble sizes were similar during Event 1 (PL/IL = 1.13, *p* = 0.349) (Figure 2C). Furthermore, during Event 1 PL/IL ratio was significantly higher in Res compared to Sus rats (*p* = 0.024). Combined, these data suggest that in male Resilient rats, the PL consistently engages a larger ensemble than IL while this PL dominance is not seen in the Susceptible rats.

It is important to stress that these differences were detected in animals who had not experienced PTSD-inducing trauma. Overall, these findings demonstrate that there were differences in the pattern of learning-induced plasticity in the mPFC between Resilient and Susceptible male rats before trauma.

### 7.2 Susceptible rats have behavioral deficits under high cognitive demand

We examined if the above-described differences in the pattern of plasticity have functional correlates by comparing behavior of Resilient and Susceptible rats in a mPFC dependent task. Figure 3A shows the RISP model and the design of Experiment 2. Details of the task are illustrated in Figure 3B.

As seen in Figure 4, The overall pattern of performance during the attentional set-shifting task was different between groups [Group × Discrimination interaction: *F*(8, 144) = 2.94, *p* < 0.005, β = 0.951] without an overall [Group effect *F*(1, 18) = 0.67, *p* = 0.423, β = 0.117]. Specifically, Resilient rats showed improvement in the reversal tasks as the training progressed, while Susceptible rats did not. Notably, Susceptible rats had significantly higher number of trails to criterion compared to Resilient rats on Reversal 3 (*p* = 0.022). There were no differences in the performance during simple and complex discriminations as evidenced by similar number of trials to criterion between groups (SD1, *p* = 0.821; SD2, *p* = 0.899; SD3, *p* = 0.587; CD, *p* = 0.087; ID, *p* = 0.884; ED, *p* = 0.758). This shows that the Susceptible male rats were able to learn and perform the simple versions of the task, but the deficits became evident under higher cognitive demand.

### 7.3 Military personnel with combat-related PTSD showed altered performance in PFC dependent task at pre-deployment evaluation

Pre-deployment cognitive evaluation of male military personnel (Figure 5A) revealed that those who developed combat-related PTSD post-deployment had a higher Cognitive Processing Time on spatial Match to Sample task (Figure 5B) compared to those who experienced combat trauma but did not develop PTSD [*F*(1, 7) = 5.906, *p* = 0.042, β = 0.550]. This difference was specific to the Match to Sample task as both groups had similar Cognitive Processing Time in other cognitive tasks such as Math [*F*(1, 7) = 0.570, *p* = 0.475, β = 0.098] (Figure 5C). Both groups also had similar Simple Reaction Time [*F*(1, 7) = 0.12, *p* = 0.916, β = 0.05] (Figure 5D). Furthermore, both groups had similar accuracy on all tasks (*p* > 0.98). The higher cognitive processing time in the Match to Sample task is consistent with functional deficits in the PFC and hippocampus and is independent of any individual differences in simple reaction time.

It is important to stress that all nine military personnel experienced severe combat-related trauma that resulted in TBI and significantly affected their post-trauma performance (not reported here as it is not relevant to the tested hypothesis).

## 8 Discussion

The set of studies reported here aimed to test the hypothesis that mPFC deficits are a putative susceptibility factor for PTSD that can be detected before trauma in both rats and humans. The results provided evidence in support of this hypothesis at both the cellular and behavioral levels in male rats and at the behavioral level in humans. At the cellular ensemble level, male rats pre-classified according to the RISP model as Susceptible to developing a PTSD-like phenotype had a different pattern of plasticity-induced IEG expression in the mPFC than Resilient rats. On the behavioral level, Susceptible rats showed deficits under higher cognitive demand before trauma. Similarly, male military personnel who later developed combat-related PTSD showed longer cognitive processing time in a PFC- and hippocampus-dependent task prior to deployment.

At the cellular level, Susceptible rats differed from Resilient rats in the relative contribution of PL and IL in spontaneous spatial learning. All rats engaged PL and IL regions of the mPFC during spatial exploration as evidenced by the presence of above-baseline number of neurons expressing plasticity-associated immediate- early genes (*Arc* and *H1a*) in both groups. However, there was a difference in the pattern of *Arc/H1a* expression between Resilient and Susceptible rats such that Resilient rats consistently engaged a larger ensemble in PL than IL while this PL dominance was not seen in the Susceptible rats. Additionally, there was a decrease in the percentage of double labeled cells in the PL of the Susceptible compared to Resilient rats. Combined with evidence from the literature where activation of PL ensembles have been linked to continuous behavioral microstates (Zhang et al., 2022) this suggests that flexible control of behavior may be diminished in Susceptible rats. The fact that these differences are present before trauma suggests that these brain regions may respond differently to a traumatic event.

The PL and IL have distinct roles in cognition and emotional regulation (Giustino and Maren, 2015; Capuzzo and Floresco, 2020). However, reciprocal connections between these brain regions allow information exchange that leads to flexible and adaptive behavior (van Aerde et al., 2008; Marek et al., 2018). Both PL and IL also receive unidirectional projections from the hippocampus (Hoover and Vertes, 2007; Sánchez-Bellot et al., 2022). and are implicated in fear learning and extinction (Quirk and Mueller, 2007; Maren et al., 2013; Giustino and Maren, 2015). Our previous data show that in these Susceptible animals the dorsal and ventral hippocampus, but not the basolateral amygdala, has altered function compared to Resilient animals before trauma (Nalloor et al., 2014). Combined with the current findings, these data suggest that susceptibility may be the product of alterations in the brain network that included the hippocampus and the mPFC. Furthermore, the hippocampus and the PFC are in brain regions that are well documented to have impaired functioning in people with established PTSD (Pitman et al., 2001; Bremner, 2007; Harnett et al., 2020). This raises the possibility that susceptible individuals develop PTSD in part due to experiencing a traumatic event differently than resilient individuals (Alexander et al., 2020).

The differences on the cellular level that are reported here (Experiment 1) were detectable because of the unique strengths offered by the *Arc/H1a* catFISH. Due to the restricted time of learning-induced *Arc/H1a* expression (< 10 min) as detected by intranuclear foci of transcription, the temporal separation of the two events (30 min), and the very low level of baseline *Arc* and *H1a* expression (< 1%), the activated neuronal ensembles are highly specific to each event. This specificity allows a more precise evaluation of changes in patterns of activation across multiple brain regions. The prominent differences in the PL and IL were not in the size of the ensembles induced by the two events but in the relative activation across the two brain regions. In addition, these findings are based on a large number of neurons per brain region and thus are representative of each region.

The differences at the cellular ensemble level were consistent with findings at the behavioral level in a hippocampal/mPFC dependent task (Attentional Set Shifting) (Birrell and Brown, 2000; Barense et al., 2002; Cernotova et al., 2021). While there were no differences in the initial learning of discrimination and reversal tasks, Susceptible rats showed impaired performance compared to Resilient rats when cognitive load increased. As the training progressed, Resilient rats improved their performance during reversals, while Susceptible rats did not. Notably, Susceptible rats started off slightly better than Resilient in the Complex Discrimination and initial Intradimensional Reversal tasks. However, they were slightly impaired in the second Intradimensional Reversal and then significantly impaired at the subsequent Extradimensional Reversal compared to Resilient rats. These findings suggest reduced baseline cognitive flexibility in male Susceptible rats. Such deficits may contribute to PTSD susceptibility by changing the experience of the trauma and anchoring it to the most aversive aspect.

The behavioral findings demonstrated in male rats paralleled those we observed in the human data set. Male military personnel who developed PTSD post-trauma had cognitive deficits that were detectable pre-trauma. Similar to rats, these differences were not universal to all tasks but were specific to a hippocampus/mPFC-dependent task (spatial Match-To-Sample) (Monk et al., 2002; Habeck et al., 2005; Schon et al., 2008; Daniel et al., 2016). The differences in cognitive processing time were not based on motivation or general inability to understand or perform the tasks, as accuracy was not affected. They were also not due to individual differences in simple reaction time, as the latter was subtracted from the individual task reaction time for each participant to derive the Cognitive Processing Time. These considerations demonstrate the specificity of the reported findings. These findings demonstrate that pre-trauma functional differences exist and can be identified in humans as well.

In addition to the strengths discussed, the study has several limitations. First, although it would have been ideal to examine alterations in patterns of plasticity during the Attentional Set-Shifting task, this was not possible due to the temporal specificity of the *Arc/H1a* catFISH method: performing the task took much longer than the time window that is optimal for inducing and detecting *Arc/H1a* intranuclear foci of transcription (3–7 min). Second, the presented data is based on studies that included only male subjects. PTSD is a sexually dimorphic disorder, so the conclusions drawn may only be applicable to males. Additional studies in female subjects are required before generalizing findings to both sexes. Third, the human data presented here is preliminary as it is based on a small sample size. Furthermore, all military personnel in this data set experienced TBI which could have influenced the PTSD diagnosis. However, the human data findings are consistent with the animal data and thus warrant larger scale study in humans to examine cognitive determinants of susceptibility to PTSD with or without physical trauma.

Despite the stated limitations, the findings suggest that mPFC deficits are a putative susceptibility factor for PTSD. In order to test whether these deficits satisfy all criteria of being a susceptibility factor, it is necessary to test if enhancing mPFC function in susceptible individuals will confer resilience to developing PTSD. This hypothesis will be addressed in future studies. Building resilience is crucial for minimizing the number of people suffering from PTSD, given that it is difficult to treat, and treatments are resource intensive and benefit only a subpopulation of people suffering from PTSD.

## Data Availability

The raw data supporting the conclusions of this article will be made available by the author, without undue reservation.
